# The identification of key genes and pathways in hepatocellular carcinoma by bioinformatics analysis of high-throughput data

**DOI:** 10.1007/s12032-017-0963-9

**Published:** 2017-04-21

**Authors:** Chaoyang Zhang, Li Peng, Yaqin Zhang, Zhaoyang Liu, Wenling Li, Shilian Chen, Guancheng Li

**Affiliations:** 1 Key Laboratory of Carcinogenesis of the Chinese Ministry of Health, Xiangya Hospital, Central South University, Changsha, People’s Republic of China; 2 Key Laboratory of Carcinogenesis and Cancer Invasion of Chinese Ministry of Education, Xiangya Hospital, Central South University, Changsha, People’s Republic of China; 3 Cancer Research Institute, Central South University, Changsha, People’s Republic of China

**Keywords:** Hepatocellular carcinoma, Bioinformatics analysis, Microarray, Differentially expressed gene

## Abstract

Liver cancer is a serious threat to public health and has fairly complicated pathogenesis. Therefore, the identification of key genes and pathways is of much importance for clarifying molecular mechanism of hepatocellular carcinoma (HCC) initiation and progression. HCC-associated gene expression dataset was downloaded from Gene Expression Omnibus database. Statistical software R was used for significance analysis of differentially expressed genes (DEGs) between liver cancer samples and normal samples. Gene Ontology (GO) term enrichment analysis and Kyoto Encyclopedia of Genes and Genomes (KEGG) pathway analysis, based on R software, were applied for the identification of pathways in which DEGs significantly enriched. Cytoscape software was for the construction of protein–protein interaction (PPI) network and module analysis to find the hub genes and key pathways. Finally, weighted correlation network analysis (WGCNA) was conducted to further screen critical gene modules with similar expression pattern and explore their biological significance. Significance analysis identified 1230 DEGs with fold change >2, including 632 significantly down-regulated DEGs and 598 significantly up-regulated DEGs. GO term enrichment analysis suggested that up-regulated DEG significantly enriched in immune response, cell adhesion, cell migration, type I interferon signaling pathway, and cell proliferation, and the down-regulated DEG mainly enriched in response to endoplasmic reticulum stress and endoplasmic reticulum unfolded protein response. KEGG pathway analysis found DEGs significantly enriched in five pathways including complement and coagulation cascades, focal adhesion, ECM–receptor interaction, antigen processing and presentation, and protein processing in endoplasmic reticulum. The top 10 hub genes in HCC were separately GMPS, ACACA, ALB, TGFB1, KRAS, ERBB2, BCL2, EGFR, STAT3, and CD8A, which resulted from PPI network. The top 3 gene interaction modules in PPI network enriched in immune response, organ development, and response to other organism, respectively. WGCNA revealed that the confirmed eight gene modules significantly enriched in monooxygenase and oxidoreductase activity, response to endoplasmic reticulum stress, type I interferon signaling pathway, processing, presentation and binding of peptide antigen, cellular response to cadmium and zinc ion, cell locomotion and differentiation, ribonucleoprotein complex and RNA processing, and immune system process, respectively. In conclusion, we identified some key genes and pathways closely related with HCC initiation and progression by a series of bioinformatics analysis on DEGs. These screened genes and pathways provided for a more detailed molecular mechanism underlying HCC occurrence and progression, holding promise for acting as biomarkers and potential therapeutic targets.

## Introduction

Liver cancer is the second leading cause of cancer associated death among men worldwide, which ranks sixth place in women over the world, suggesting that liver cancer is more common in men than in women [1]. In China, liver cancer is the fourth most commonly diagnosed cancer in men, and the estimated new cases and deaths are 466.1 thousands and 422.1 thousands, respectively, in 2015 [2]. The most common type of liver cancer is hepatocellular carcinoma (HCC). The risk factors for liver cancer include chronic hepatitis B virus (HBV) and hepatitis C virus (HCV) infection, consumption of food such as corn and peanuts polluted by aflatoxin, obesity, type 2 diabetes, heavy alcohol consumption-associated cirrhosis, and smoking [3–5]. At present, the commonly used treatment approaches of liver cancer include surgical resection, radiotherapy, chemotherapy, and targeted therapy to improve patients’ prognosis and recurrence. Nevertheless, the 5-year survival rate of HCC is still low, especially in advanced HCC [6]. In addition, most patients miss the optimal treatment period because of no significant clinical symptoms at early stage of HCC. Consequently, it needs more effort to clarify the molecular mechanism underlying HCC development and progression, holding promise for finding potential drug targets and diagnostic biomarkers of HCC.

Gene expression analysis based on microarray technology is a widely used, high-throughput and powerful research method, which can simultaneously detect expression change of thousands of genes on the mRNA level. By gene expression profiling analysis with microarray technology, some investigations have found many differently expressed genes which played a critical role in HCC initiation and progression and could be assessed as potential molecular targets and diagnostic markers. Zhai et al. [7] proved that HOXC10 could function as a important mediator of invasion in cervical cancer by means of gene expression analysis. Through gene expression profiling, Sato et al. [8] demonstrated that epigenetic modification including promoter hypermethylation and histone deacetylation were the leading cause of the down-regulation of CDKN1C. In the current study, we identified HCC-associated DEGs between cancerous and normal samples, and successively performed GO term enrichment analysis, KEGG pathway analysis, PPI network analysis and gene co-expression network analysis to discover the key genes and pathways closely related to HCC.

## Methods and materials

### Acquisition of microarray data

Gene Expression Omnibus (GEO, http://www.ncbi.nlm.nih.gov/geo/) database in the National Center for Biotechnology Information (NCBI) is used to store curated gene expression datasets, original series and platform records. Hepatocellular carcinoma-associated dataset GSE14323 submitted by Kellie J. Archer and based on GPL571 platform ([HG-U133A_2] Affymetrix Human Genome U133A 2.0 Array), was downloaded from GEO database, including 38 HCC samples and 19 normal samples [9].

### Identification of DEGs

Statistical software R (version 3.3.2, https://www.r-project.org/) and packages of Bioconductor (http://www.bioconductor.org/) were applied to significance analysis of DEGs between HCC samples and normal samples. At first, quality detection on microarray data was successively conducted by quality control overview diagram, weights and residuals plot, relative log expression (RLE) box plot, normalized unscaled standard errors (NUSE) box plot, RNA degradation curve, principal components plot (PCA) and clustering analysis diagram based on “simpleaffy,” “affyPLM,” “RColorBrewer,” “affy,” “gcrma,” “graph,” and “affycoretools” packages, to remove unqualified samples [10, 11]. Then, integrative algorithm “gcRMA” was chosen for preprocessing of microarray data [12]. Empirical Bayes method was used to select significant DEGs based on “limma” package of Bioconductor [13]. Finally, DEGs were annotated by “annotate” package. A *P* < 0.05 was considered statistically significant.

### GO term and KEGG pathway enrichment analysis

Biological significance of DEGs was explored by GO term enrichment analysis including biological process, cellular component and molecular function, based on Bioconductor packages “GOstats.” KEGG pathway enrichment analysis of DEGs was performed by Bioconductor packages “GeneAnswers” to find critical pathways closely related to HCC initiation and progression. A *P* < 0.05 was considered to have statistical significance and to achieve significant enrichment.

### Protein–protein interaction (PPI) network analysis

PPI network can help us identity the key genes and important gene modules which are involved in HCC development from interaction level. PPI information of DEGs was acquired from Search Tool for the Retrieval of Interacting Genes (STRING) database (http://www.string-db.org/). Then, Cytoscape software was used for construction of PPI network. At last, module analysis and GO analysis were carried out by two plug-ins Molecular Complex Detection (MCODE) and Biological Network Gene Ontology tool (BiNGO) in Cytoscape to illuminate the biological significance of gene modules in HCC. *P* value less than 0.05 was considered significantly different.

### Weighted correlation network analysis of DEGs

As a system biology method, gene co-expression network analysis was performed by weighted correlation network analysis (WGCNA) package to describe the correlation of gene expression pattern and to screen highly correlated gene modules, holding promise for finding candidate biomarkers and drug targets [14]. In this co-expression network, nodes represented DEGs, and the correlation of gene expression pattern was defined as connectivity degree among genes [15]. In brief, excessive missing value and outlier microarray samples were firstly detected according to DEGs expression matrix. The soft thresholding power was determined by analysis of network topology. Gene co-expression similarity and adjacency were successively calculated using the soft thresholding power. Then, the adjacency was transformed into topological overlap matrix (TOM). Finally, hierarchical clustering was conducted using TOM and the dynamic tree cut algorithm was applied to modules screening, after which we performed GO enrichment analysis on gene modules to characterize modules related to HCC.

## Results

### The identification of DEGs

**Fig. 1:**
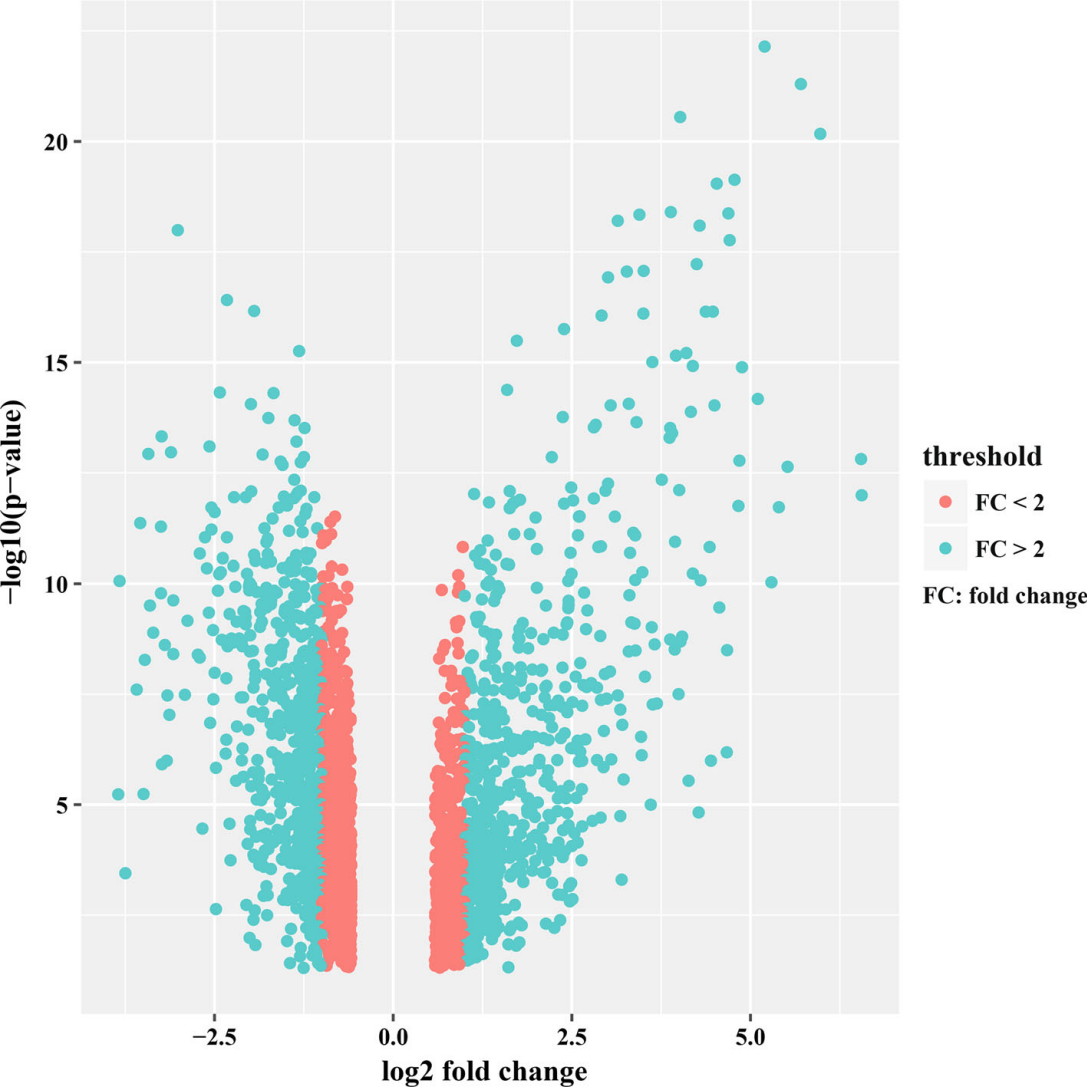
Volcano plot of 2546 DEGs. *Red* DEGs with fold change <2; *turquoise* DEGs with fold change >2

**Fig. 2:**
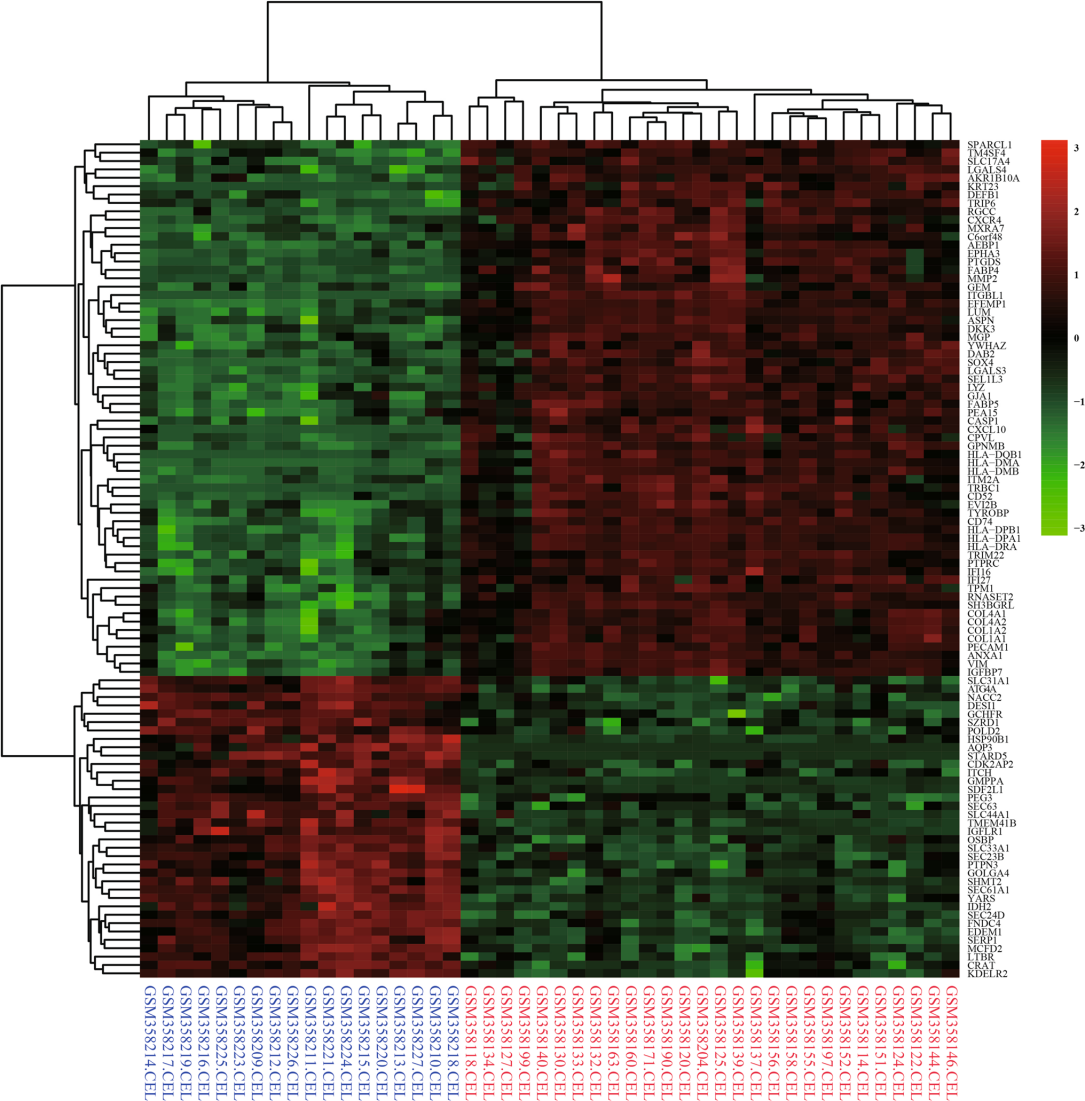
Heatmap of the top 100 DEGs with fold change >2. *Red* up-regulated DEGs; *green* down-regulated DEGs

Gene expression dataset GSE14323 was downloaded from GEO database. After quality detection of microarray raw data, we removed 11 unqualified microarrays and retained the rest of 46 microarrays based on GPL571 platform, including 28 HCC samples and 18 normal samples. Statistical analysis software R was used for preprocessing and gene differential expression analysis of microarray data. There were altogether 2546 DEGs, among which we selected 1230 DEGs (fold change >2, Fig. 1) consisting 632 significantly down-regulated DEGs and 598 significantly up-regulated DEGs, for the subsequent bioinformatics analysis. The expression level of the top 100 DEGs with fold change >2 was displayed in Fig. 2.

### GO term enrichment analysis of DEGs

**Fig. 3:**
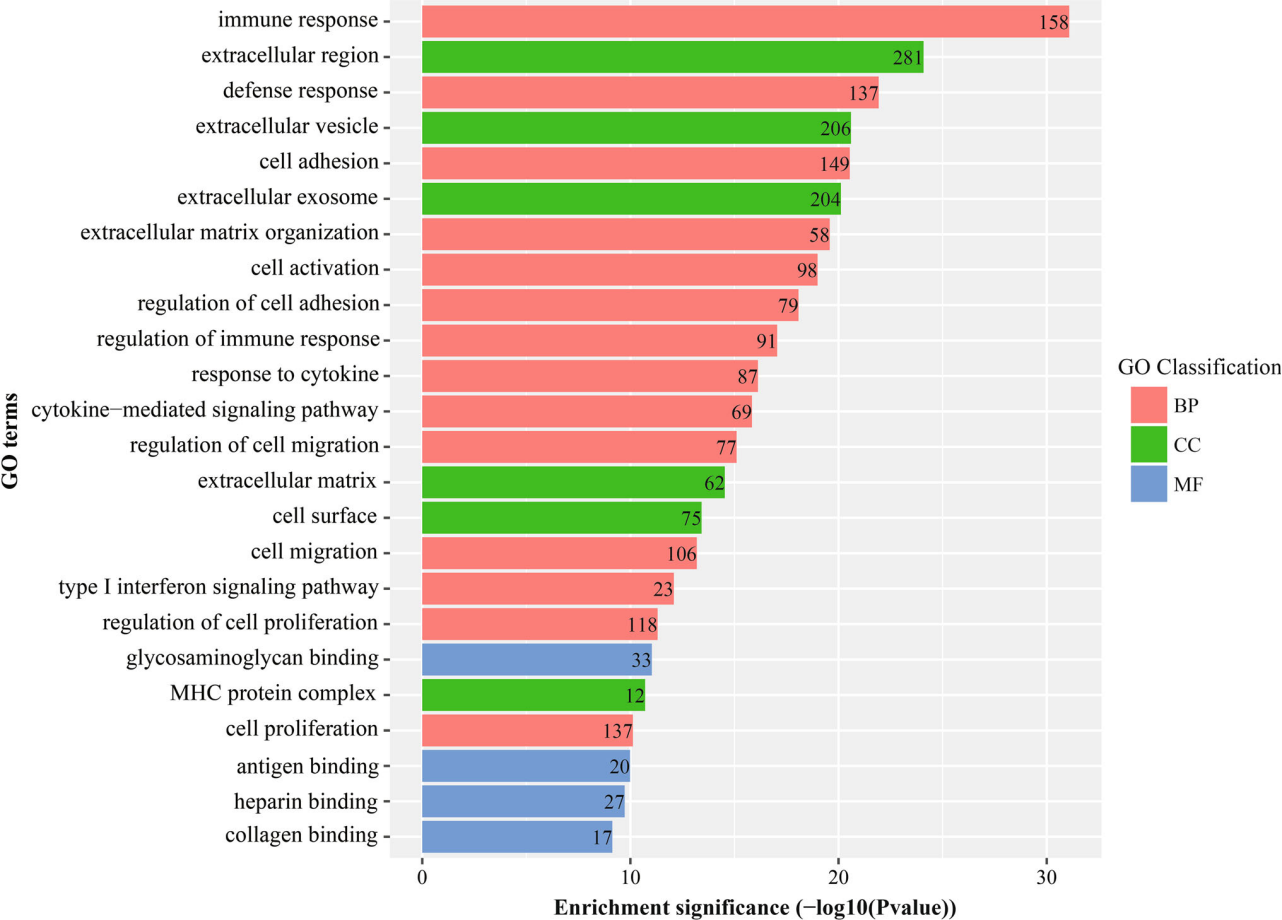
GO enrichment analysis result of up-regulated DEGs with fold change >2. *BP* biological process, *CC* cellular component, *MF* molecular function

**Fig. 4:**
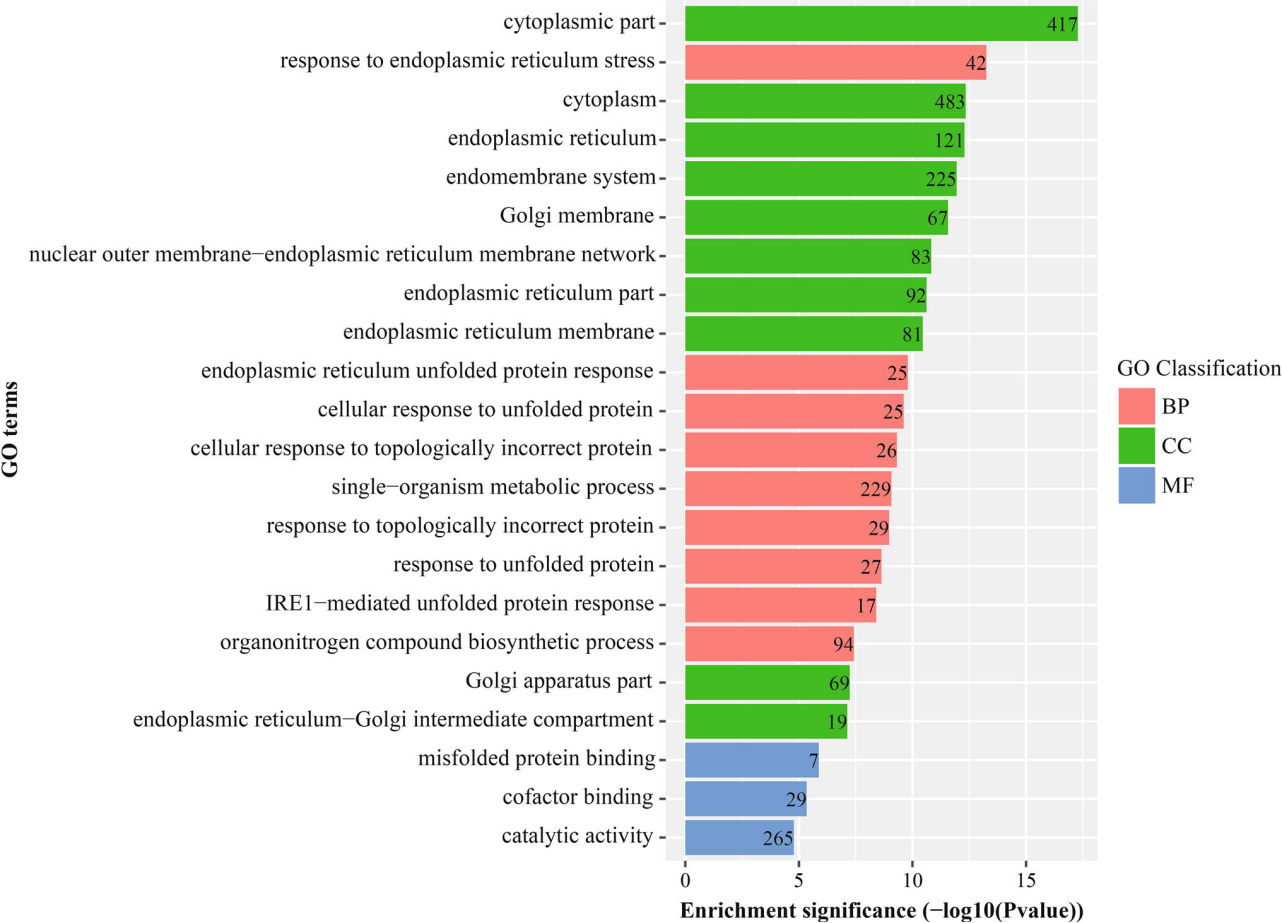
GO enrichment analysis result of down-regulated DEGs with fold change <2. *BP* biological process, *CC* cellular component, *MF* molecular function

GO term enrichment analysis results varied from GO classification and expression change of DEGs. As to biological process, the up-regulated DEGs significantly enriched in immune response, defense response, cell adhesion, cell migration, type I interferon signaling pathway and cell proliferation, and the down-regulated DEGs significantly enriched in response to endoplasmic reticulum stress, endoplasmic reticulum unfolded protein response and cellular response to unfolded protein. For cellular component, the up-regulated DEGs significantly enriched in extracellular region, extracellular vesicle, extracellular exosome, cell surface and MHC protein complex, and the down-regulated DEGs significantly enriched in cytoplasmic part, endoplasmic reticulum, endomembrane system, and Golgi membrane. About molecular function, the up-regulated DEGs significantly enriched in glycosaminoglycan binding, antigen binding, heparin binding, and collagen binding, and the down-regulated DEGs significantly enriched in misfolded protein binding, cofactor binding and catalytic binding. More detailed GO enrichment analysis results are shown in Figs. 3 and 4. These significantly enriched pathways and terms could help us a lot to further understand the role which DEGs played in HCC occurrence and progress.

### KEGG pathway analysis of DEGs

**Fig. 5:**
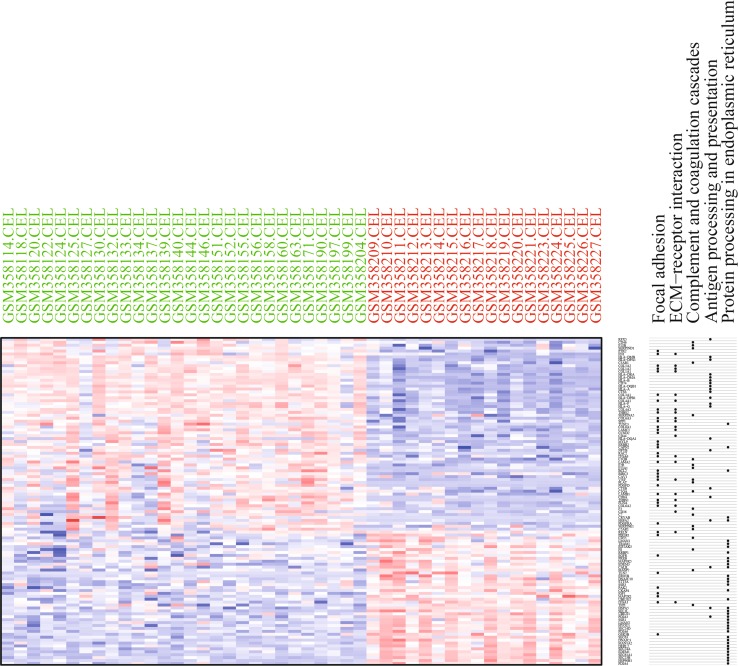
Heatmap of DEGs significantly enriched KEGG pathways. *Red* up-regulation; *blue* down-regulation

As shown in Fig. 5, KEGG pathway analysis found five significantly enriched pathways. Fifteen up-regulated DEGs and four down-regulated DEGs enriched in complement and coagulation cascades. Thirty up-regulated DEGs and nine down-regulated DEGs enriched in focal adhesion. Twenty up-regulated DEGs and two down-regulated DEGs enriched in ECM–receptor interaction. Eighteen up-regulated DEGs and three down-regulated DEGs enriched in antigen processing and presentation. Five up-regulated DEGs and 32 down-regulated DEGs enriched in protein processing in endoplasmic reticulum. This analysis results was obviously different from GO terms enrichment analysis, indicating fairly complicated molecular mechanism existing in HCC.

### Protein–protein interaction network analysis of DEGs

**Fig. 6:**
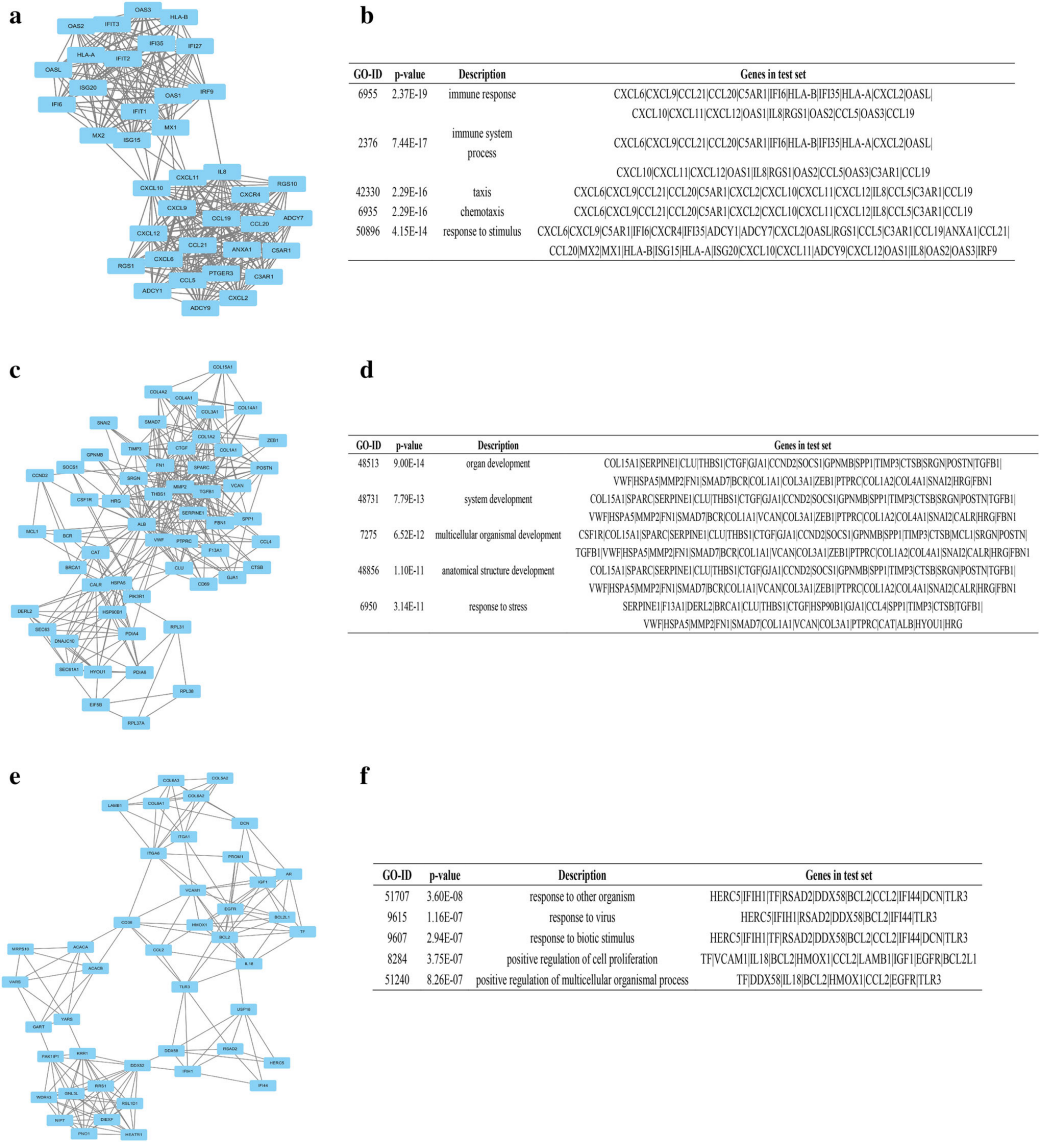
Module analysis of PPI network. **a** module 1; **b** GO enrichment analysis of module 1; **c** module 2; **d** GO enrichment analysis of module 2; **e** module 3; **f** GO enrichment analysis of module 3

Protein–protein interaction (PPI) network of DEGs, consisting of 1072 nodes and 7155 edges, was constructed by Cytoscape software, based on STRING database. The top 10 DEGs with high degree of connectivity were selected as the hub genes of HCC. These hub genes were separately guanine monophosphate synthase (GMPS), acetyl-CoA carboxylase alpha (ACACA), albumin (ALB), transforming growth factor beta 1 (TGFB1), KRAS proto-oncogene, GTPase (KRAS), erb-b2 receptor tyrosine kinase 2 (ERBB2), BCL2, apoptosis regulator (BCL2), epidermal growth factor receptor (EGFR), and signal transducer and activator of transcription 3 (STAT3) and CD8a molecule (CD8A), which might play a critical role in HCC progression. Two plug-ins MCODE and BiNGO were used to carry out module analysis in Cytoscape software. The top three gene modules were significantly enriched in immune response, organ development, and response to other organism, respectively (Fig. 6).

### Weighted correlation network analysis of DEGs

**Fig. 7:**
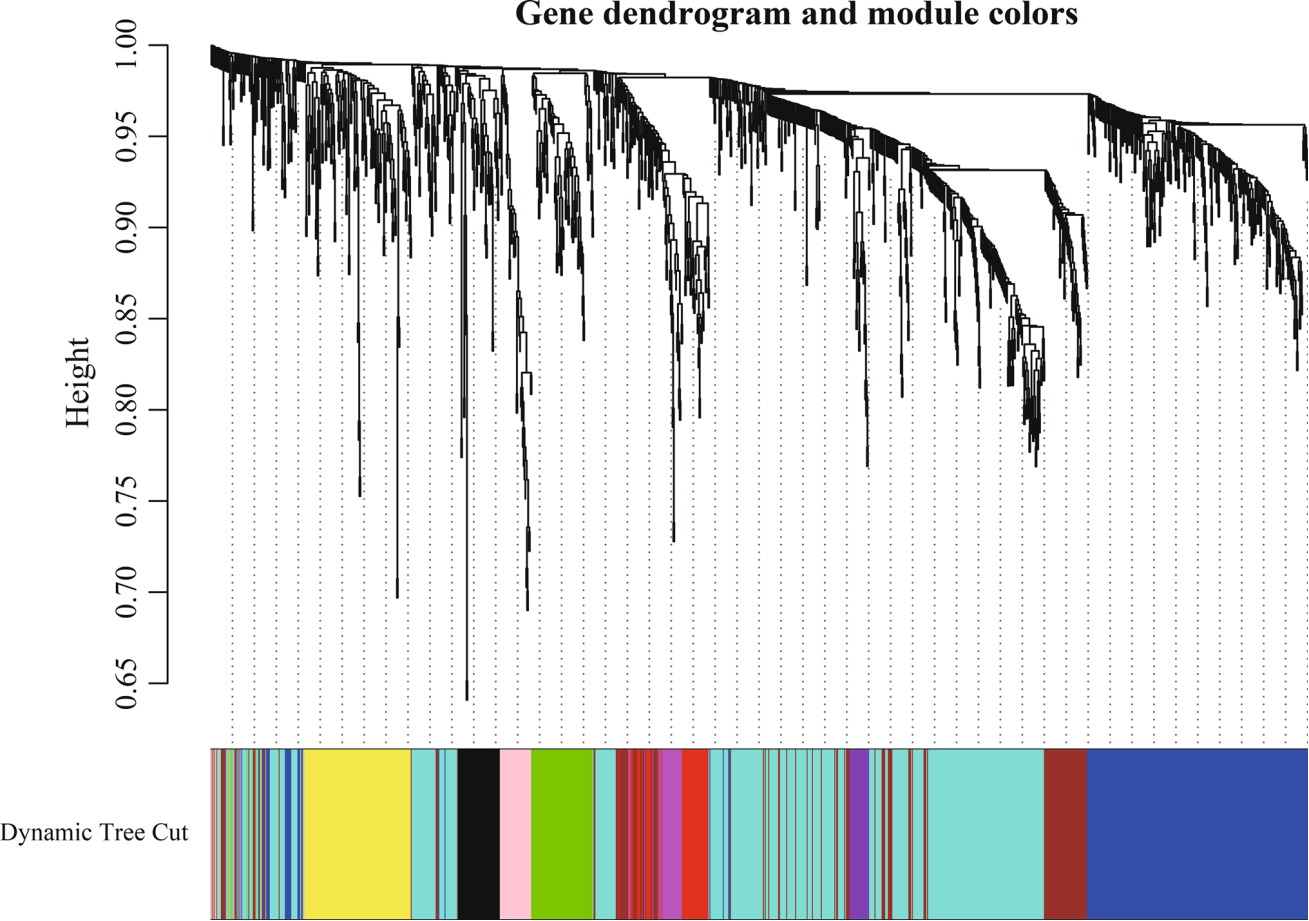
DEGs clustering and module screening based on gene expression pattern. The *top* was gene dendrogram and the *bottom* was genes modules with different colors

**Fig. 8:**
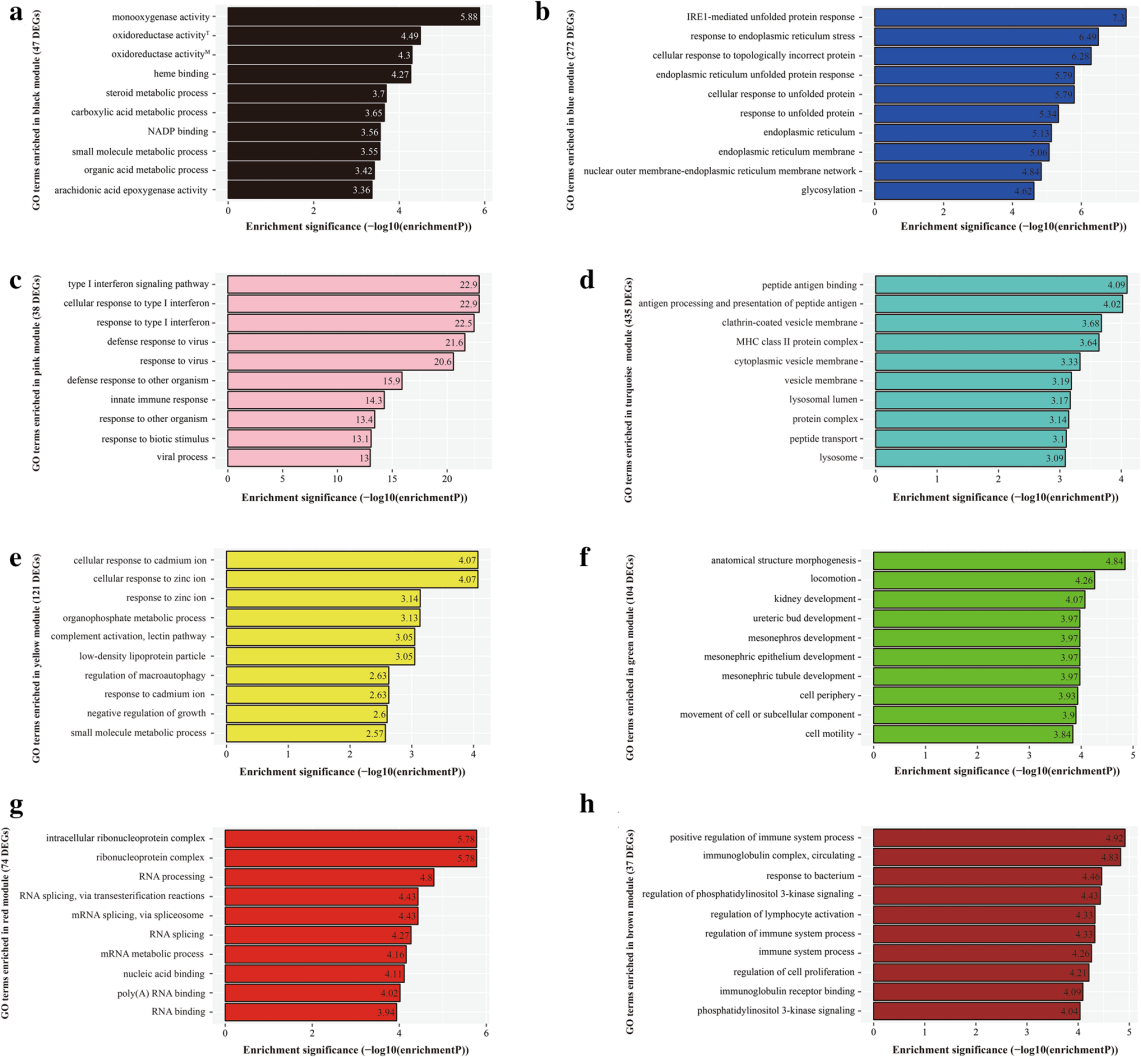
GO enrichment analysis of 8 genes modules. **a** black module, superscript “T” represented “oxidoreductase activity, acting on paired donors, with incorporation or reduction of molecular oxygen”; superscript “M” represented “oxidoreductase activity, acting on paired donors, with incorporation or reduction of molecular oxygen, NAD(P)H as one donor, and incorporation of one atom of oxygen”; **b** blue module; **c** pink module; **d** turquoise module; **e** yellow module; **f** green module; **g** red module; **h** brown module

Genes with relationship of regulation or interaction tend to display same or similar expression pattern. Consequently, we constructed the DEGs co-expression network to screen gene modules with similar expression profile. In total, we screened eight gene modules as shown in Fig. 7 through DEGs cluster analysis and dynamic tree cut algorithm. Each color represented one gene module including a certain number of DEGs with similar expression pattern. GO enrichment analysis on gene module was performed to find the key modules and biological processes closely related to HCC. As we can see in Fig. 8, specifically, DEGs in black module significantly enriched in monooxygenase activity and oxidoreductase activity; DEGs in blue module significantly enriched in IRE1-mediated unfolded protein response and response to endoplasmic reticulum stress; DEGs in pink module significantly enriched in type I interferon signaling pathway and cellular response to type I interferon; DEGs in turquoise module significantly enriched in peptide antigen binding and antigen processing and presentation of peptide antigen; DEGs in yellow module significantly enriched in cellular response to cadmium ion and cellular response to zinc ion; DEGs in green module significantly enriched in anatomical structure morphogenesis and locomotion; DEGs in red module significantly enriched in intracellular ribonucleoprotein complex and ribonucleoprotein complex; DEGs in brown module significantly enriched in positive regulation of immune system process and immunoglobulin complex, circulating.

## Discussion

In the present study, we identified significant DEGs between cancerous and normal samples and conducted a series of bioinformatics analysis to screen key genes and pathways closely related to HCC. By significance analysis on microarray data in statistical software R, we identified 1230 DEGs with fold change over 2, including 632 up-regulated DEGs and 598 down-regulated DEGs. Bioinformatics analysis on DEGs including GO term enrichment analysis, KEGG pathway analysis, PPI network analysis and WGCNA found HCC-associated genes and pathways, which exerted momentous effect on cancer initiation and progression from different sides.

Immune response and HCC. By comprehensive analysis, we found all the bioinformatics analysis results mentioned immune response-related genes and pathways. Specifically, GO term enrichment analysis showed that 158 up-regulated DEGs significantly enriched in immune response and 91 up-regulated DEGs enriched in regulation of immune response. KEGG pathway analysis indicated that 18 up-regulated DEGs and 3 down-regulated DEGs significantly enriched in antigen processing and presentation. In addition, 15 up-regulated DEGs and 4 down-regulated DEGs enriched in complement and coagulation cascades. PPI network and module analysis found that the first gene module significantly enriched in immune response. Co-expression network analysis by WGCNA suggested that pink module including 38 up-regulated DEGs significantly enrich in type I interferon signaling pathway. Consequently, we could initially conclude that many DEGs participated in immune response including antigen processing and presentation, complement cascades, and type I interferon signaling pathway to influence liver inflammation response and liver cancer progression. Most of HCC incidence results from chronic infection of HBV or HCV, and inflammatory response deriving from chronic infection contributes to HCC development, while immunotherapy of HCC aims to activate and promote immunity, prompting that immune response is closely related to HCC progression [16]. Type I interferon, including IFN-α and IFN-β, can directly inhibit the proliferation and facilitate the apoptosis of tumor cell, or indirectly regulate tumor microenvironment [17, 18]. The up-regulation of type I interferon signaling pathway could result from lymphocyte response to the high-dose infection of HBV or HCV, and displayed the complicated interaction mechanism between type I interferon mediated immunosuppression and liver cell carcinogenesis. Therefore, more effort is needed to confirm the relationship between up-regulation of type I interferon and HCC progression. As a part of innate immune system, complement system was traditionally supposed to play a suppressive role on the tumor occurrence and development. In recent years, however, there were increasing evidence demonstrating that complement component and complement activated product can promote tumor cell growth, tumor angiogenesis, and immunosuppression [19–24]. The up-regulation of complement cascades in our study supported the latest investigations. Tumor antigen processing and presentation, including MHC I pathway and MHC II pathway, assist CD8 T cell to kill target cells and CD4 T cell to generate cytokine to activate other immune cells, respectively. HCV infection into HCC samples could be the leading cause of the increased ability of antigen processing and presentation.

Endoplasmic reticulum (ER) stress and HCC. GO term enrichment analysis suggested that 42 down-regulated DEGs significantly enriched in response to ER stress and 25 down-regulated DEGs enriched in ER unfolded protein response. KEGG pathway analysis found that five up-regulated DEGs and 32 down-regulated DEGs significantly enriched in protein processing in endoplasmic reticulum. WGCNA indicated that blue module including 272 down-regulated DEGs, mainly enriched in IRE1-mediated unfolded protein response (URP) and response to ER stress. ER stress response, induced by the accumulation of the unfold protein in the ER, can lead to the activation of inositol-requiring transmembrane kinase/endonuclease-1 (IRE1) to further increase the expression level of URP related gene and promote unfold protein response to maintain cellular homeostasis [25]. Some researches have confirmed that chronic ER stress existed in diverse cancers and played a important role in tumor cell growth and apoptosis [26–28]. In the present study, down-regulation of the majority of DEGs meant reduction in ER stress response and unfold protein response, prompting that liver cancer cell in currently specific tumor stage has accomplished cellular homeostasis to avoid apoptosis induced by continuous ER stress response. Therefore, accelerating cell apoptosis by elevating the expression of ER stress-associated genes to bring out enduring ER stress response could function as a promising therapeutic strategy of HCC.

Cell adhesion and HCC. According to GO term enrichment analysis results, 149 up-regulated DEGs significantly enriched in cell adhesion, and 79 up-regulated DEGs enriched in regulation of cell adhesion. On the basis of KEGG pathway analysis results, 30 up-regulated DEGs and 9 down-regulated DEGs enriched in focal adhesion. Besides, 20 up-regulated DEGs and two down-regulated DEGs enriched in ECM-receptor interaction. As we all know, cancer metastasis is the pivotal cause of tumor patient deaths [29]. The capture and adhesion of cancer cell in microcirculation are the first condition of cancer metastasis through vessel [30, 31]. Accordingly, overexpression of cell adhesion molecules could enhance focal adhesion and ECM-receptor interaction and accelerate liver cancer cell metastasis in vessel and settlement in metastatic sites. In metastatic HCC or advanced HCC, targeting cell adhesion molecules might be a potentially effective therapeutic method.

In addition to these pathways we have discussed above, GO term enrichment analysis also suggested that up-regulated DEGs positively participated in HCC initiation and development through cell migration and cell proliferation. Modules analysis in PPI network found that the top three gene modules primarily enriched in immune response, organ development and response to other organism, which was consistent with what we have discussed in the above. The top 10 hub genes were GMPS, ACACA, ALB, TGFB1, KRAS, ERBB2, BCL2, EGFR, STAT3, and CD8A, respectively. With the highest degree of connectivity in PPI network, ALB combined with bilirubin, namely albumin–bilirubin grade, displayed a higher prognostic value than Child–Pugh grade in HCC patients, suggesting that ALB was a good prognostic biomarker of HCC [32]. GMPS, a crucial enzyme of de novo purine biosynthesis, has been identified as an important p53 repression target by proteomic analysis, and its up-regulation led to disruption of tumor-suppressive p53 network in liver cancer [33]. Consequently, GMPS was a pivotal contributor to HCC progression and could function as a potential therapeutic target to maintain the stability of p53 network. The overexpression of EGFR plays a positive role in progression of HCC by contributing to cell proliferation, migration and invasion, and EGFR has been demonstrated a relatively effective drug target and a good prognostic biomarker [34–37]. As a downstream regulatory object of EGFR in EGFR-STAT3 oncogenic pathway, aberrantly activated and up-regulated STAT3 signaling pathway has been detected in various cancers including HCC and is considered as an essential risk factor for tumor initiation and development [38–40]. However, in our study, both EGFR and STAT3 in HCC samples were significantly down-regulated, which might be responsible for EGFR-targeting therapy failure in some HCC cases. The discrepancy of EGFR–STAT3 pathway expression in different studies suggested these oncogenes displayed spatial and temporal-specific expression and also reflected tumor heterogeneity in HCC. Hence the more effective molecular-targeting therapy should be based on specific gene expression profiling of each patient in consideration of individual difference.

Co-expression network analysis by WGCNA found eight gene modules with highly relevant expression pattern. Then GO term enrichment analysis was conducted to explore the biological significance of each gene module. Black module including 47 DEGs mainly was involved in redox reaction of cancer cell by regulating monooxygenase and oxidoreductase activity. Redox imbalance, which results from disruption of the homeostasis of endogenous antioxidants and oxidants and is expressed as elevated oxidative stress, has been confirmed in cancer cell [41]. In order to avoid cell apoptosis and growth arrest, various cancer cells have formed a series of antioxidant mechanisms to remove excessive oxidants and sustain stable redox status [42, 43]. Blue module including 272 DEGs were closely related to IRE1-mediated unfolded protein response (URP) and response to endoplasmic reticulum (ER) stress. Pink module including 38 DEGs was significantly enriched in type I interferon signaling pathway which has been discussed in GO term enrichment analysis results, prompting the crucial significance of immune response associated pathways in HCC development. Turquoise module including 435 DEGs principally was involved in processing, presentation, and binding of peptide antigen, which were several key biological processes in adaptive immune response. Yellow module including 121 DEGs participated in cellular response to cadmium and zinc ion. Green module including 104 DEGs was correlated with cell locomotion and differentiation, which was consistent with GO term enrichment analysis results. The up-regulated DEGs associated with cell locomotion could facilitate liver cancer cell migration and invasion to contribute to HCC progression. Red module including 74 DEGs was enriched in ribonucleoprotein complex and RNA processing. Brown module including 37 DEGs took part in immune system process and regulation of immune system process, further verifying the close relationship of immune response and HCC, as we have mentioned in GO enrichment and KEGG pathway analysis. In brief, the confirmed eight gene modules were involved in different biological processes to play an important role in HCC initiation and progression from various aspects.

## Conclusion

In conclusion, we identified some key genes and pathways closely related with HCC initiation and progression by a series of bioinformatics analysis on DEGs between HCC samples and normal samples. For example, immune response including antigen processing and presentation, complement cascades and type I interferon signaling pathway participated in HCV carcinogenic process and contributed to HCC development. These identified genes and pathways provided for a more detailed molecular mechanism underlying HCC occurrence and progression, holding promise for acting as potential biomarkers and therapeutic targets.
